# Participant perceptions of HAT TRICK™ Cricket: a culturally-adapted intervention for men with South Asian backgrounds in Australia

**DOI:** 10.1093/heapro/daag001

**Published:** 2026-01-23

**Authors:** Nico Schulenkorf, Sinan Koparan, Madeleine English, Paul Sharp, Hugh Sixsmith, Lauren M Wood, Patrick Farhart, Cristina M Caperchione

**Affiliations:** Business School, Sport Management, University of Technology Sydney, Gadigal Land, Eora Nation, Sydney, New South Wales 2007, Australia; Centre for Sport Leadership, Stellenbosch University, Private Bag X1, Stellenbosch 7599, South Africa; School of Sport, Exercise and Rehabilitation and Human Performance Research Centre, University of Technology Sydney, Gadigal Land, Eora Nation, Sydney, New South Wales 2007, Australia; School of Sport, Exercise and Rehabilitation and Human Performance Research Centre, University of Technology Sydney, Gadigal Land, Eora Nation, Sydney, New South Wales 2007, Australia; School of Health Sciences, University of New South Wales, Sydney, Gadigal Land, Eora Nation, Sydney, New South Wales 2007, Australia; School of Sport, Exercise and Rehabilitation and Human Performance Research Centre, University of Technology Sydney, Gadigal Land, Eora Nation, Sydney, New South Wales 2007, Australia; School of Sport, Exercise and Rehabilitation and Human Performance Research Centre, University of Technology Sydney, Gadigal Land, Eora Nation, Sydney, New South Wales 2007, Australia; School of Sport, Exercise and Rehabilitation and Human Performance Research Centre, University of Technology Sydney, Gadigal Land, Eora Nation, Sydney, New South Wales 2007, Australia; Cricket New South Wales, Sydney, Gadigal Land, Eora Nation, Sydney, New South Wales 2007, Australia; School of Sport, Exercise and Rehabilitation and Human Performance Research Centre, University of Technology Sydney, Gadigal Land, Eora Nation, Sydney, New South Wales 2007, Australia

**Keywords:** culturally adapted, co-design, social connectedness, physical activity, cricket, community health promotion

## Abstract

Individuals from culturally and linguistically diverse (CALD) backgrounds frequently experience complex health barriers arising from migration related factors and cultural differences within their new communities. Men with South Asian backgrounds in Australia represent a group particularly affected by these intersecting factors in addition to gender-related influences. In response, HAT TRICK™ Cricket, a culturally adapted health promotion intervention aimed at improving physical activity, healthy eating, and mental fitness, was designed for men with South Asian backgrounds in Western Sydney, Australia. To explore participants’ perspectives and experiences of the programme and its perceived outcomes on their health and well-being, semi-structured interviews (*N* = 13) were conducted approximately 2 weeks following programme completion. Three themes were inductively derived using thematic analysis: ‘Pursuing personal growth and mastery on and off the pitch’ which emerged through opportunities for experiential learning, culturally meaningful engagement, and skill development that collectively enhanced participants’ confidence, sense of competence, and motivation to improve; ‘Finding commonality and social connection’ in which participants perceived the programme to foster meaningful interactions, facilitate the formation of new friendships, and strengthen existing relationships in a supportive environment that encouraged open and honest conversations extending beyond sport; and third, ‘Translating knowledge into everyday action’ which indicated that participants actively applied some of the skills and knowledge gained from the programme to improve their daily physical activity, nutrition, and mental health practices. These findings support the feasibility and value of culturally tailored sport-based health promotion programmes and can inform future initiatives for CALD communities.

**Contribution to Health Promotion**

- The study’s findings highlight the importance of ‘layered learning’ in health promotion programmes, where knowledge is built progressively through expert input, peer engagement, and reflective practice.
- The programme focus, design features, nutrition content, and role models featured a distinct South Asian touch and reinforce the value of co-designed, community-informed interventions that centre the lived experiences and cultural contexts of participants.
- This approach ensures that health promotion is not only relevant but also empowering, particularly for men from migrant backgrounds navigating complex intersections of culture, masculinity, and health.

## Background

Australia is universally recognized for its increasingly multicultural identity, with nearly half (48.2%) of the population reporting at least one parent born overseas, and more than a quarter (27.6%) of its inhabitants born overseas themselves (Australian Bureau of Statistics 2023). While diversity enriches Australian society, migration can also present unique challenges that affect the health and well-being of individuals from culturally and linguistically diverse (CALD) communities. Practical and structural barriers such as language difficulties, limited health literacy, financial constraints, unfamiliarity with the healthcare system, and lack of culturally responsive services can limit access to health promoting services (Harrison *et al*. 2020, Khatri and Assefa 2022). These challenges are further compounded by broader post-migration stressors, such as social isolation, disrupted support networks, and unfamiliar cultural norms (Castañeda *et al*. 2015, Mak *et al*. 2021, Shea and Wong 2022).

The South Asian community, comprising individuals from India, Sri Lanka, Pakistan, Bangladesh, and Nepal, has emerged as one of the fastest-growing migrant groups in Australia (Australian Bureau of Statistics 2023). India became the second most common country of birth outside Australia with an increase of 467 000 people since 2013, representing the largest population growth among all overseas born groups (Australian Bureau of Statistics 2023). This demographic shift has drawn attention to the distinct health profile of South Asian Australians, who exhibit a higher mean chronic disease risk index compared to the Australian-born population (Sarich *et al*. 2015). Notably, immigrants with South Asian backgrounds have a greater prevalence of type 2 diabetes and cardiovascular disease (Gupta *et al*. 2015, Australian Institute of Health and Welfare 2023) than other Australians (Australian Institute of Health and Welfare 2023). As such, tailored interventions focusing on modifiable risk factors for chronic diseases, such as physical activity (PA) and healthy eating, are needed.

In responding to this need, consideration of gender is critical as culturally linked gender norms in male immigrants with South Asian backgrounds further compound barriers to health. Cultural models of masculinity emphasizing strength, self-reliance, and emotional restraint can discourage men from acknowledging illness, engaging in preventative care, or participating in community-based health initiatives (Fikree and Pasha 2004, Daluwatta *et al*. 2022). The experience of migration can also disrupt traditional protector and provider gender roles and collectivist values, adding stress and identity challenges that may further isolate men or prevent them from prioritizing their health (Oliffe *et al*. 2009, Kukreja 2021). In this context, health promotion strategies must not only be culturally relevant but also engage critically with the gendered dimensions of health behaviour. Programmes that create supportive, male-friendly environments are essential to reaching and sustaining the involvement of men with South Asian backgrounds in meaningful health interventions.

One promising approach is sport-based health promotion, which uses sport as an accessible and culturally meaningful vehicle for engagement (Schulenkorf and Siefken 2019, Timm *et al*. 2025). However, there has been limited historical focus on culturally diverse male cohorts with regards to gender-sensitive health promotion programmes and sporting opportunities (Nguyen *et al*. 2024, Hodder *et al*. 2025). Within South Asian communities, cricket holds particular cultural significance and can serve as a familiar and appealing platform to deliver health interventions (Shafqat and Bharucha 2004, Sheikh *et al*. 2013, Hylton and Long 2024). Sport-based programmes not only promote PA but also enhance language skills, social connection, and a sense of belonging (Theeboom *et al*. 2023, Karstensen *et al*. 2024, Lange *et al*. 2024), all of which are vital for health and successful integration into Australian society.

Building on those insights, HAT TRICK™ Cricket was developed as a culturally adapted health promotion programme specifically designed for men with South Asian backgrounds in Australia. The initiative draws from the success of the previous HAT TRICK™ programme, which used team sports such as ice hockey to engage men in lifestyle changes and health education (Sharp *et al*. 2020, Caperchione *et al*. 2021). By leveraging cricket, a sport deeply embedded in South Asian identity, the programme aims to improve physical health, provide targeted health education, and foster stronger community ties within a culturally safe environment. This study explores the experiences of men with South Asian backgrounds participating in HAT TRICK™ Cricket and its perceived outcomes to better understand how culturally tailored sport-based interventions can influence health behaviours, improve psychosocial well-being, and foster a greater sense of social connectedness.

### Setting and context

In brief, the established HAT TRICK™ programme is a 12-week group-based intervention focused on PA, diet, and social connectedness for inactive, overweight men. Weekly 90-minute sessions comprised of 45 minutes of PA participation (e.g. strength training, sport, walking) and 45 minutes of health education (e.g. how to accumulate daily PA, understanding macronutrients, managing symptoms of stress), while simultaneously promoting increased social connectedness through ‘man-friendly’ interactive and informal style of learning. A ‘Playbook’ resource manual is also provided to further supplement health education topics. The intervention is theory-guided, drawing on multiple constructs from the Social Cognitive Theory (Bandura 1986) and Self-Determination Theory (Ryan and Deci 2017, Teixeira *et al*. 2020, Ryan 2023) and on health models and theories which incorporate gender and socially constructed masculinities (Creighton and Oliffe 2010) as an important social determinant. Full details of the HAT TRICK™ programme and design features have been published elsewhere (Caperchione *et al*. 2017, 2021).

Through an iterative co-design process (Vargas *et al*. 2022), the original HAT TRICK™ was culturally adapted in collaboration with community partners (i.e. state and local cricket organizations) and end-users (i.e. men with South Asian backgrounds from the community) to create HAT TRICK™ Cricket. Built upon principles of respect and shared ownership, the co-design process involved a series of meetings to define the programme’s scope and engage participants in collaborative problem solving and iterative design. HAT TRICK™ Cricket continued to comprise of 12 weekly, 90-minute sessions with a focus on cricket-based activities and culturally adapted health education. Health education (approx. 30 minute/session, rather than 45 minutes) aimed to improve health literacy and awareness of PA and healthy eating, as well as offer practical strategies for improving mental fitness (i.e. an adaptable process leveraging skills and resources to maintain well-being) and social connectedness. Health education was followed by 60 minutes of cricket-based PA (rather than 45 minutes), such as batting or bowling in the cricket nets, cricket-related games, or strength and conditioning sessions run by qualified facilitators. This adjustment in time was made based on feedback from end-users, who noted that men with South Asian backgrounds would be more likely to engage with a ‘cricket-first’ format. The reduced time allocated to health education did not impact discussion topics, rather these discussions were innovatively embedded and further elaborated on during the cricket-based PA or in the ‘Playbook’ (outlined below). For instance, while playing cricket participants discussed how movement and being active can assist with reducing stress.

Further, and upon request from industry stakeholders and end-users, the programme also included select modules from the Volunteer Certification Programme (VCP) developed by the governing body of cricket in New South Wales, Cricket, NSW. This component aimed to promote capacity building and confidence of community members to coach or umpire at a local club, thereby enhancing opportunities for social integration and community engagement through cricket.

All participants were provided with a programme manual (i.e. the ‘Playbook’) which contained practical strategies and tools to introduce healthy lifestyle behaviours into the participants’ daily routines. Key changes from the original manual included the replacement of sporting role models with more culturally relevant cricket icons (e.g. Brett Lee, Virat Kohli, and Sachin Tendulkar) and modifications to the nutrition content to better align with traditional South Asian diets (e.g. sugar content in Masala Chai) and further elaboration on topics previously addressed during the health education component (e.g. ways to incorporate incidental PA during your day). Additionally, design features such as colours, layout templates, diagrams, and pictures were adapted in consultation with stakeholders and end-users during the co-design phase. Throughout the programme, guest experts including a dietician of South Asian background working within the South Asian community, sports psychologists, former professional cricket players, and professional cricket coaches were invited to share their knowledge. Overall, the delivery of the HAT TRICK^TM^ Cricket programme promoted enjoyment and social connectedness, from a culturally appropriate lens and on male-preferred methods of communication such as honest and realistic messaging, humour, and positive banter (Sharp *et al*. 2025a, 2025b).

## Methods

### Research design

This exploratory study is underpinned by an interpretivist mode of inquiry which suggests that access to reality can be socially constructed through language, consciousness, and shared meanings (Glesne 1999, Neuman 2021). Specifically, interpretivist research acknowledges that data are analysed through a process of induction, denoting that the researchers construct and reconstruct meaning in relation to the research question and based on the realities of participants (Denzin *et al*. 2024). In other words, interpretivist studies aim to understand the context of a phenomenon through the meanings that people assign to it.

### Participant recruitment

Participants (*n* = 13) were men who had completed the 12-week HAT TRICK™ Cricket intervention. Participants were recruited from Western Sydney, an area of Sydney in which 40% of the population is born overseas, with the majority of these individuals hailing from India. All participants self-identified as Indian. Participant characteristics are outlined in Table 1. Purposeful sampling was used to select men who attended at least 50% of the programme sessions, ensuring they could provide insights across a range of programme components. This well-established qualitative approach facilitates the selection of individuals who are able to provide rich and relevant information (Palinkas *et al*. 2015, Sharp *et al*. 2018). Eligible participants were contacted by email and invited to take part in a one-on-one online interview at a mutually convenient time. A total of 18 men were contacted; 5 did not respond, resulting in 13 interviewees who accepted the invitation.

**Table 1:** Participant characteristics.

Characteristic	Participants^a^ (*n* = 13)
Age	
25–29	1
30–34	1
35–39	2
40–44	6
45–49	1
Country of birth	
India	10
United Arab Emirates	1
Country of birth parent 1	
India	11
Country of birth parent 2	
India	11
Length of involvement in Cricket Club	
Not previously involved	1
1–2 seasons	5
3–4 seasons	2
5–6 seasons	2
7+ seasons	1

^a^Two participants did not provide demographic characteristics.

### Data collection

In this study, semi-structured online interviews were used to explore participants’ perceptions of and experiences with HAT TRICK™ Cricket, focusing on perceived benefits, challenges and outcomes from participation. Zoom interviews were chosen as they can provide rich qualitative data and are time- and cost-effective (Archibald *et al*. 2019, Oliffe *et al*. 2021). Participants were also given the option to complete interviews by telephone in case they did not feel comfortable using the online interface. One participant opted for a telephone interview. Ethical approval was obtained by the University of Technology Sydney Human Research Ethics Committee (#ETH23-8496).

A semi-structured interview guide was developed to explore participants’ perspectives and experiences of participating in HAT TRICK™ Cricket. Throughout the interview process, member reflection was employed through clarifying questions and seeking feedback on the alignment of initial interpretations with participants’ perspectives (Tracy 2010, Braun and Clarke 2024). All interviews were conducted by the lead male programme facilitator and research assistant (H.S.) who worked with the men throughout the programme and has experience in qualitative data collection. Written informed consent was obtained prior to the scheduled interviews and renewed verbally at the time of the interview. Interviews lasted approximately 30 minutes, were audio recorded, and transcribed using the Zoom software recording and transcription functions. Transcriptions were reviewed for accuracy, and all identifiable information was removed to ensure anonymity and confidentiality following transcription.

### Data analysis

The interpretivist paradigm that underpins this study highlights that empirical findings should inform the codes, themes, and patterns which are generated from the research. In other words, categories should not be entirely imposed prior to data collection but themes should develop inductively through a qualitative data analysis process that can be described as ‘working with data, organising it, breaking it into manageable units, synthesizing it, searching for patterns, discovering what is important and what is to be learned, and deciding what you will tell others’ (Bogdan and Biklen 1982).

The management software used to support the reflexive thematic data analysis process was NVivo14™ which assisted the researchers in integrating, indexing, and coding the data. The process followed current recommendations (Braun *et al*. 2016, Braun and Clarke 2021) for reflexive thematic analysis and was undertaken in six phases: (i) familiarization with the data and recognition of patterns, (ii) creation of codes, (iii) generation of initial themes, (iv) development and review of themes, (v) refining, defining, and naming themes, and (vi) writing up. As part of this process, each of the interview transcripts were read several times by two trained researchers (S.K. and N.S.) to allow for an in-depth discussion of the data, coding, and interpretation. Specifically, S.K. and N.S. independently coded and categorized the various responses before codes with overlapping properties were collated into three newly generated themes. As an example, initial coding resulted in the sub-themes ‘Meeting likeminded people’, ‘Networking’, and ‘Social interaction’ which were subsequently merged into the final theme ‘Finding commonality and social connection’. The final themes are presented below, with descriptive summaries and data extracts (i.e. quotations) to support these themes. Pseudonyms have been used to anonymize participants’ identities.

To enhance methodological rigour and congruency, the quality practice by way of ‘critical friends’ was utilized. This practice encourages reflexivity and provides an opportunity for co-researchers to challenge interpretations and offer critical feedback during the analysis and writing process (Smith and McGannon 2018, Braun and Clarke 2024). During these discussions (with critical friends), researchers reflected on their own positionalities and considered how their gender, cultural backgrounds (i.e. European and Australian ancestry and upbringing), personal experiences with cricket, and relationships within South Asian communities may have shaped their interpretations of data. For example, the research team includes a male individual of European background who has a basic understanding of cricket as a sport, but he spent many years as a sport volunteer and academic researcher in Sri Lanka. His lived experiences of local traditions and customs and the ability to interpret programme preferences including the design, set-up, implementation, and certification of activities differed from other members who can be described as Australian cricket enthusiasts but with limited on-the-ground experience in South Asia. Overall, the reflective dialogue among the research team helped individuals develop a deeper critical consciousness, enhancing awareness of how their social positions and assumptions impacted the research process, including the interpretation and analysis of findings and its outcomes (Pillen *et al*. 2020).

## Findings

HAT TRICK™ Cricket was specifically tailored to men with South Asian backgrounds and was designed to leverage the sport of cricket for physical, social, and mental health benefits. Overall, the interviews indicated that participants viewed the culturally adapted HAT TRICK programme and its content as relevant, engaging, and culturally resonant. Specific findings are presented via three overarching themes that reflect participants’ experiences of participation and perceived outcomes, including (i) ‘Pursuing personal growth and mastery on and off the pitch’, (ii) ‘Finding commonality and social connection’, and (iii) ‘Translating knowledge into everyday action’.

### Pursuing personal growth and mastery on and off the pitch

HAT TRICK™ Cricket offered a range of valuable learning opportunities for participants. Selected sessions were led by sport and health experts who shared professional knowledge and experience relevant to their areas of specialization. These expert-led sessions enabled participants to engage deeply with key programme focus areas including the development of psychological skills, physical performance, technical development, and nutritional health. When reflecting on the session led by a professional cricket player, Ram highlighted:

Yeah, he was helpful with my leg spin. But [more importantly], he guided me that I’m better off doing off-spin, because that would be a more natural action for me. With my action and the kind of shoulder that I have, there’s a great chance that I’m not able to bowl in line.

These reflections suggest that participants’ initial motivation to enhance their cricket skills provided a culturally familiar and personally meaningful entry point into the programme, which in turn opened space for discussions that extended beyond sport to include physical and mental health preparations in competitive settings. For example, Nish reflected:

So, when I play now, that mindset is important for me to perform. Because that is the difference between when you just play in the nets and you are hitting the shots everywhere, and [when it matters] on the ground where that special mindset has to be there.

In addition to on-field training, the sessions were also designed to relate to participants’ personal lives off the field. Specifically, the programme’s focus on self-development was a critical feature of HAT TRICK™ Cricket as all participants had the opportunity to acquire socio-psychological skills where they learnt about mental focus, goal setting, motivation, and self-confidence in the context of cricket. Ram commented on the session conducted by a sport psychologist:

I would have never known the psychological part about how to get mentally prepared when you go to bat or when you go to bowl. What processes do you follow?! [But] during the programme, I tried them, and I could see value in them. And I just wanted to learn more about the lifestyle part of [the game].

Finally, participants also indicated transfer of their developed socio-psychological skills beyond cricket and into their personal lives. For example, Rayyan explained that he kept using some of the mental techniques developed through the programme in his daily routines:

Oh, yeah, the mental health sessions [were critical]. I forgot what that exercise was called, but it’s the one with the little green circles. I’ve used that a couple of times afterwards [at home]. You know, it really helps, not just from a sport perspective, from an overall personal level as well.

This statement demonstrates how performance-oriented learning served as a bridge to health-related reflection, as men connected mental preparation and self-awareness in cricket to broader ideas of managing challenges in daily life.

In addition to sessions focused on professional and personal development, HAT TRICK™ Cricket integrated elements of Cricket NSW’s VCP which included coaching and umpiring modules. The VCP was intended to provide exposure to activities beyond playing (i.e. umpiring and coaching), and participants used these opportunities to enhance their own understanding of the game. For example, they were able to address uncertainties in their understanding of the rules, recognizing that their interpretations were not always aligned with official or intended meanings.

Through their involvement in both the umpiring and coaching courses of the certification programme, participants had the opportunity to discuss challenges directly with experts, gaining a clearer understanding of the rules and their application. Ram shared his learning experiences around dismissals and the empowering nature of the overall advice he received:

The [sessions] were so valuable, definitely, I learned a few bits about umpiring. [VCP facilitator] debunked so many myths and that was very good to know. Like I always have had issues with the LBWs [Leg Before Wickets]. So, she gave me very simple and easy tips and that would at least make me get 70%, right. Which is good enough. [The sessions] would help me figure things out! I was always scared about giving LBWs, like, I didn’t know how that worked.

These responses demonstrate that the culturally tailored programme created a welcoming and familiar space that supported participants’ desire for sporting and personal growth. A key factor driving their engagement was the opportunity to improve themselves through cricket and broader life skills. In short, participants perceived the expert-led sessions as valuable learning opportunities that signalled personal growth and development, increasing the acceptability and value of attending a healthy lifestyle intervention.

### Finding commonality and social connection

HAT TRICK™ Cricket created an opportunity for participants to engage meaningfully with one another. Since participants attended the programme as members of their respective clubs, it offered a chance to strengthen community feelings by connecting outside of regular club settings and during the offseason. In other words, it allowed for a deepening of relationships in a more social environment. Further to this, sessions included multiple clubs which facilitated interactions among participants beyond an intra-club environment. Biraj highlighted the significance of being able to openly communicate and engage with peers around sport and social issues:

It was exciting to be able to talk about the sport that you love and something that you are really passionate about without anybody judging you… It’s like, you know, Double A meetings. So, it’s a cricket support group, if you want to call it that, which is good.

Biraj’s appreciation to talk freely about the sport he loves and the comparison to Alcoholics Anonymous (AA) meetings highlight how the programme created an inclusive, safe space where open and honest conversations were welcomed—and where aspects beyond sport, including social and mental support, became a key factor. This depth of emotional safety did not come out of nowhere but was achieved through a combination of carefully curated activities and expert facilitation, which provided opportunities for participants to exchange ideas and to share personal experiences. For example, facilitators were trained to move away from deficit ‘shame and blame’ based discussions and adopt a strength-based approach that encouraged participants to openly discuss behaviours often perceived as ‘failures’ or ‘bad habits’ in a more constructive way (similar to AA). Through activities such as goal-setting and collaborative problem-solving, participants worked together to explore underlying challenges and identify positive, practical solutions for behaviour change. In addition, the health education component of the programme included ice-breakers, team building tasks, and group discussions on various aspects of well-being (e.g. mental fitness, dealing with setbacks and recruiting a support system), supporting reflection and social connection. As such, all participants were encouraged to engage with their peers, and many formed new connections and friendships during the programme. For example, Adesh stated: ‘Oh, yes, absolutely! I have got a few new friends whom I didn’t know [before]’ and Emad highlighted: ‘I have new connections with the team. Some of them I knew from before but others I’ve connected with for the first time there.’

Dhruv noted that some of the friendships that originated during HAT TRICK™ Cricket actually lasted beyond the programme:

Yeah, there were people who I have stayed connected with post-cricket. For example, we needed certain players for our club as well and so this programme gave me that opportunity to reach out and connect with those people.

The ability to stay connected long-term goes beyond the immediate benefit of meeting new people; however, it should be acknowledged that newly formed friendships at the programme do not automatically extend into people’s daily lives. For example, Ram stated:

I made good connections with some of the fellow mates. We exchanged numbers as well. We haven’t been keeping in touch that much. But at least I know someone that I can always talk to.

In addition to the forming of new relationships, the most significant social benefit that participants identified was the ability to learn from each other. This was made possible by the group-oriented nature of the programme which encouraged collaboration, discussion, and sharing of diverse perspectives. Engaging in group discussions and completing PA components as a group allowed participants to exchange practical insights, techniques, and personal experiences, all of which contributed to individual skill development and personal growth. This notion is illustrated by Adesh:

Basically, it’s always learning from each other. It’s more of experience sharing and it’s more of the interaction. Knowledge goes both ways, both for me as well as for the other person. So definitely, it is because of the group [setting].

Adesh’s sentiments are reinforced by Dev who highlighted the benefits of a reciprocal learning environment where participants not only gained insights from peers but also contributed their own knowledge. This related to social as well as sporting experiences:

To give a small example, I bowl off spin. So, when I was just bowling in the nets, I had people who were giving me some tips, so that was voluntary. And [later] I was able to share some tips of feedback with the other people who were with me. So, it was more of a give and take and interactions which may be small, but valuable.

The interviews highlighted that on many occasions, participants constructively pointed out areas for improvement in each other’s technique and offered tailored suggestions, fostering a collaborative environment focused on mutual growth, skill development, and assisting each other. Here, the social learning experience complements the previously discussed professional learning benefits generated by engaging with the expert-led training sessions. Furthermore, learning from others was not limited to the confines of scheduled sessions. Meaningful learning also occurred informally, as Ram recalled:

There used to be some discussions after the programme where we would just stand in the parking lot and talk a little bit. There was a session that we had with [peers’ names] and all of us were just talking - I was telling them about my epiphanies and my issues with batting and all those things, and they were guiding me. Can you try this? Can you try that? That was really helpful. There were some really good questions asked by [peer’s name] for me to think about.

Ram’s reflection highlights the value of these informal exchanges which occurred naturally and spontaneously outside the official sessions. In short, these moments provided additional opportunities for participants to engage with, and learn from, their peers by sharing experiences, insights, and guidance.

### Translating knowledge into everyday action

HAT TRICK™ Cricket demonstrated clear translational outcomes as participants indicated that they utilized the knowledge and skills acquired beyond the programme itself. In fact, they applied their enhanced understanding of PA, exercise, and nutrition to improve their performance not only on the field but also beyond the cricket pitch. Expert tips for integrating PA into daily living were particularly appreciated by participants for their simplicity and accessibility, as outlined by Dev:

Some tips [were excellent]. For example, I don’t have a car. I generally use public transport, so I was encouraged to get off a stop before and then I walk. So those kinds of tips were given. And if I’m doing some exercise that was beneficial, I try to get a partner and do it together, one pulls the other. That was one of the tips provided there. These small, small tips really helped.

This reflection illustrates how behaviour change was facilitated when programme content aligned with participants’ everyday realities and resources. Practical, achievable strategies, such as walking rather than structured exercise, resonated with men’s preference for autonomy and action-oriented approaches, making the guidance both acceptable and sustainable. HAT TRICK™ Cricket participant Moh also indicated that he utilized the exercises and warm-ups more broadly beyond the programme:

There were some exercises that were taught that I now do on a regular basis. Yeah, especially one of those warm-ups that [Facilitator Name] led. That really helped us understand how to have a warm-up, how I have to open up my shoulders and all before I do anything on the field, so those I continue to do now.

This example highlights how using sport-specific activities as the foundation for learning about movement and fitness provided a culturally and socially acceptable entry point for men to engage with health promotion. By framing physical activity within the familiar language of cricket, the programme normalized exercise as part of sporting mastery rather than as a separate or medicalized behaviour change goal.

To assist with the uptake and upkeep of PA, participants were provided with a Playbook at the start of the programme. Participants highlighted the value of this resource in reinforcing key messages supporting independent reflection and practice beyond the sessions. For example, Emad stated:

Yes, I have skimmed through it a couple of times, looked into certain aspects of it, tried some of those stretches and exercises and stuff like that. And I’ve started involving my kid also, my son, and he also started enjoying it. Perhaps not on a regular basis, but… whenever I get time, I do the stretching. And he also joins me. He is also interested in seeing what I am doing, and then mimicking those. And then I correct him. Okay, this is how you should do that and stuff. It’s enjoyable. Yeah.

Emad’s reflection underscores the ease with which some of the participants incorporated PA suggestions from the programme into their everyday routine. Moreover, Emad’s initiative to involve his child extends the impact of the programme beyond the individual, suggesting that the tips and exercises were effective, shareable and engaging for others.

In addition to the PA knowledge gained by participants, the men highlighted greater awareness and knowledge concerning nutrition. Biraj explained how attending the dietitian session prompted greater attention to his eating habits: ‘After the dietitian session, I have been carefully observing what I’m eating, how much I’m eating and what changes I need to make.’ Importantly, participants described integrating this information into their daily routines and sporting preparation, reframing healthy eating as essential fuel for energy, focus, and performance both on and off the pitch. Many also commented on the usefulness of the additional and supplementary nutritional information within the Playbook, referring to the section about healthy food swaps. Ultimately, the combination of the expert-led session and the Playbook helped to increase participants’ awareness and understanding of how to develop and maintain healthy nutritional habits. It also encouraged reflection on existing eating habits and supported informed changes and strategies to improve them. These outcomes may well be a result of several key mechanisms including the credibility and relatability of the dietitian, the use of practical and culturally familiar food examples (e.g. fruit chaat, dahl, and roti), and the availability of the Playbook as a tangible resource that reinforced learning outside the sessions. Together, these elements enabled participants to connect new knowledge to their own routines and make gradual, sustainable adjustments.

Further, participants reported being more conscious when purchasing products, often taking the time to read and evaluate product labels before making purchasing decisions. For example, Dhruv stated:

Yeah, definitely. So, every day now when I go to Woolworths or Coles, I look at the ingredients. I wasn’t looking at those earlier. So yeah, that’s one of those changes the programme definitely had.

Emad added that he used his newly gained knowledge to inform decision-making in his household, and share his insights with friends and family:

Since that session and I’ve started focusing on okay ‘I should be looking at these aspects when I pick things from the store or the shop.’ And then back at home, [I speak] to my kids. You can’t stop the kids from eating [unhealthy food], but at least to an extent I’ve cut down on certain things. Whatever I learned, I’ve tried to pass it on to the family as well, and to friends also. I’ve told them that these are the things that we shouldn’t be using and promoting.

This example, representative of a number of participant responses, illustrates the translation of programme knowledge into everyday action, where participants integrated new practices within their households and social networks. It reflects the extension of learning beyond the programme environment and how participants applied and sharing knowledge in ways that influence daily habits and family routines. Participants’ efforts to actively share insights with their families and social circles further support the translational impact of the programme.

## Discussion

The HAT TRICK™ Cricket programme was specifically tailored to men with South Asian backgrounds and designed to leverage the sport of cricket for health and social benefits. Several interrelated factors contributed to participants’ positive perceptions of the programme, offering important lessons for practitioners and academics interested in employing sport and PA initiatives in CALD communities.

HAT TRICK™ Cricket effectively positioned the cricket pitch as both a literal and metaphorical space of learning. Through structured sessions led by subject matter experts (i.e. professional cricketers, dietitians, and sports psychologists), participants were exposed to high-quality, contextually relevant knowledge spanning physical, psychological, and social health domains. This multidimensional approach aligns with existing literature on the criticality of embedding health literacy within culturally resonant frameworks (Hunt *et al*. 2014, Caperchione *et al*. 2021). Participants clearly articulated how guidance from these experts went beyond the game of cricket and provided opportunities to gain knowledge and understanding of psychological preparedness and healthy lifestyle strategies. The integration of expert insights into their daily routines, ranging from improved cricket skills and techniques to cognitive strategies for performance, demonstrates a successful translation of theory into practice. Importantly, the opportunity to build cricket skills functioned as a culturally resonant engagement strategy that allowed for conversations about health-related behaviour change. This finding supports previous evidence that sport-based interventions can serve as effective vehicles for delivering health and well-being education, particularly for men who are otherwise reluctant to engage with traditional health promotion avenues (Bottorff *et al*. 2015).

Equally critical was the emergent peer-to-peer learning culture that developed throughout the programme. The collaborative and dialogical structure of HAT TRICK™ Cricket fostered a mutual learning environment where participants could share, receive, and apply feedback organically. These interactions extended beyond the formal sessions, occurring in informal contexts like car parks post-session. Such unscripted exchanges may be particularly impactful, as they reflect a deeper cultural engagement with learned behaviours and ideas, consistent with social learning theory (Bandura 1977) and adult learning principles (Knowles *et al*. 2011).

Participants’ descriptions of sharing tips, offering feedback, and problem-solving collectively reflect the type of relational accountability that is essential for sustainable behaviour change (Stewart *et al*. 2023, Hibbin 2024). These findings challenge assumptions that men are reluctant to discuss their health. In this case, the cricket-based format allowed participants to reframe vulnerability and inquisitiveness as part of team camaraderie and skill development, rather than weakness. This subtle shift is critical in promoting help-seeking and sustained engagement in health-promoting behaviours (Addis and Mahalik 2003, Gough 2007) and aligns with research on masculine help-seeking practices, which suggest that men are more likely to engage in discussions about mental health when these occur within familiar, activity-based settings (Sharp *et al*. 2022). Within HAT TRICK™, the cricket-based format and peer interaction allowed participants to reframe help-seeking and inquisitiveness as part of teamwork and skill development, rather than as signs of weakness. This recontextualization of vulnerability within masculine norms of competence and collaboration represents a critical mechanism through which sport-based programmes can support sustained engagement and positive health behaviours among men (Sharp *et al*. 2025a, 2025b).

One of the most salient achievements of the programme was its role in cultivating social connectedness among participants—a benefit that was both immediate and, for some, sustained. Participants made it clear that the programme operated not merely as a physical health intervention, but as a psychosocial support network. In doing so, HAT TRICK™ Cricket functioned as what Bourdieu (1986) termed a ‘social field’ in which trust, recognition, and shared values are generated and mobilized. This achievement is particularly important given that many men with South Asian and migrant backgrounds are often grappling with the psychosocial stressors of resettlement, marginalization, and cultural dissonance. In such circumstances, the absence of culturally familiar, non-stigmatizing social spaces can exacerbate feelings of isolation (Daluwatta *et al*. 2022, Prajapati and Liebling 2022). HAT TRICK™ Cricket, by contrast, provided a culturally safe and normatively appropriate space in which men could engage authentically and free from judgment. As such, our study supports previous research in the health promotion and sport-for-development space which has highlighted the importance of safe and non-judgemental spaces for priority populations and disadvantaged and marginalized communities across physical, psychological, socio-cultural, political and experimental domains (Spaaij and Schulenkorf 2014, Garner-Purkis *et al*. 2020, Timm *et al*. 2025).

As an extension to social connectedness, the programme also enhanced bridging and bonding of social capital. In line with Putnam’s *et al*. (1993, 2003) understanding of social capital as a public good which requires participation as a core element to bind people and communities together, bonding capital was observed through deeper intra-club relationships, while bridging capital emerged from interactions with members of other clubs and leagues. These new connections often transcended the programme itself, facilitating ongoing communication, networking, and even player recruitment. While not all participants maintained these connections, their existence affirms that HAT TRICK™ Cricket extended the boundaries of individual social networks.

Importantly, these outcomes speak to the multidimensional value of community sport beyond physical health. Similar to other purposefully designed sport-for-development or sport-for-health initiatives (Schulenkorf 2017, Edwards and Rowe 2019, Schulenkorf and Siefken 2019), HAT TRICK™ Cricket reconfigured sporting environments to serve as platforms for social integration and mental well-being. For instance, the deliberate inclusion of group discussions centred around collective problem-solving tasks created a supportive environment where participants could share their experiences with past health and sport challenges, successes, and lessons learned. This collective approach not only encouraged mutual assistance but also helped to break down stigma and judgment. As a result, participants felt more comfortable sharing personal experiences, thereby enhancing their sense of connectedness and reducing social isolation, two factors that are known to improve mental health outcomes and reduce health disparities among populations of migrant background (Sharp *et al*. 2020, Tran *et al*. 2022, Nwofe *et al*. 2024).

The success of HAT TRICK™ Cricket hinged not just on its delivery but on its thoughtful planning, co-design, and integration of holistic health components. The programme’s use of cricket, a sport deeply embedded in both Australian and South Asian cultural identities, served as an effective Trojan horse for embedding discussions on health, well-being, and social engagement. This cultural congruence ensured the programme’s acceptability and increased the likelihood of sustained engagement, echoing evidence from previous gender-sensitized, sport-based interventions (Gray *et al*. 2013, Sharp *et al*. 2021).

From a design perspective, HAT TRICK™ Cricket adopted an integrated and holistic approach by intentionally combining physical activity, health education, and mental fitness components. This design bridged the traditional separation of these domains within men’s health promotion, addressing their interconnection through a culturally meaningful sport-based context. Consequently, participants not only improved physical fitness but some of them adopted healthier eating behaviours and psychological strategies in both sport and everyday life—an achievement that speaks to the translational benefits of purposeful programme design. Specifically, PA routines, warm-up techniques, food label literacy, and mental focus exercises were absorbed and applied in daily life and often shared with family members. Such diffusion of knowledge suggests an active ripple effect in health behaviour change (Sugden 2006, Nobles *et al*. 2022), expanding beyond the individual participant to influence broader family and community systems.

In addition to underpinning co-design principles and a clear focus on cultural relevance and acceptability, the integration of the VCP components such as umpiring and coaching were beneficial for community participants. Specifically, they broadened the scope of engagement and allowed for capacity building. These additions reframed participants not just as recipients of knowledge but as future contributors and leaders within their cricketing communities. This upskilling approach represents a fundamental step in the sustainable scaling of programmes like HAT TRICK™ Cricket—akin to a train-the-trainers approach (see Gustin *et al*. 2016, Schulenkorf and Sugden, 2011) where participants transition from learners to facilitators, multiplying the programme’s impact and increasing critical community ownership (Hoekman *et al*. 2019). However, sustainable development requires longitudinal vision. While the short-term translational outcomes are promising, questions remain about the durability of these changes without continued scaffolding. This points to the importance of ongoing community support, refresher sessions, and integration with broader health infrastructure to reinforce and extend the gains made. Designing feedback loops and follow-up components into the programme model could ensure a continuum of engagement and support.

## Strengths and limitations

This explorative qualitative study provides a nuanced understanding of HAT TRICK™ Cricket participants’ experiences and perceived outcomes. From a methodological perspective, a number of limitations must be considered. First, while all participants could speak English, it was not the first language for many. As such, some were less confident in articulating their thoughts, which may have limited the depth and clarity of certain responses. Furthermore, while the 2-week follow-up period was beneficial for participants’ recall abilities, the study is limited in its ability to comment on the programme’s sustained, long-term impact on key outcomes. Another potential limitation is social desirability bias, given the interviewer’s dual role as the former programme facilitator, which may have influenced participants’ openness. However, this role overlap also offered benefit as it established rapport and fostered trust with participants, thus enabling deeper discussions. Moreover, the familiarity with the programme allowed for more targeted and insightful probing, including efforts to encourage candid reflections and emphasizing the value of honest feedback for future programme improvements.

## Conclusion

HAT TRICK™ Cricket was tailored for men with South Asian backgrounds and designed to leverage the sport of cricket for physical, social, and mental health benefits. It represents an inclusive and promising approach to health promotion for men from migrant backgrounds, combining expert instruction, peer-based learning, and psychosocial support in a culturally resonant, enjoyable, and professional format. It enabled participants to navigate and reconcile culturally complex understandings of masculinity, competitiveness, and health. Moreover, it bridged gaps in health literacy, addressed social isolation, and promoted help seeking in a non-stigmatizing way. These findings highlight the necessity of layered learning experiences in gender-sensitized programmes and further reinforce the value of co-designed, community-informed interventions. Future interventions could build upon this model by integrating longitudinal and sustainable support mechanisms, exploring scalability across other cultural groups and sporting contexts, and embedding evaluation frameworks to track long-term outcomes.

## Data Availability

Due to identifiable details within transcripts and the study’s small sample size, the authors have chosen not to publicly share data in order to protect participants’ anonymity and confidentiality.
